# Stress and Well-Being Intervention and mHealth Delivery Adaptation for Latinx Millennial Caregivers: Qualitative User-Centered Design Approach

**DOI:** 10.2196/73621

**Published:** 2025-10-06

**Authors:** Megan Thomas Hebdon, Galilea D Dupree, Janice Hernandez, Heather Cuevas, Michael Thomas, Shane Burt, Neil Peterson, Sharon D Horner

**Affiliations:** 1 College of Nursing, University of Utah, 10 S. 2000 E., Salt Lake City, UT, United States, 1-801-581-3414; 2 Dell Seton Medical Center Austin, Austin, TX, United States; 3 School of Nursing, University of Texas, Austin, TX, United States; 4 College of Nursing, Brigham Young University, Provo, UT, United States; 5 Moses H Cone Memorial Hospital, Greensboro, NC, United States

**Keywords:** Latinx, family caregiver, millennial, mobile health, mHealth, user-centered design

## Abstract

**Background:**

This study aimed to adapt a stress and well-being intervention delivered via a mobile health (mHealth) app for Latinx millennial caregivers. This demographic, born between 1981 and 1996, represents a significant portion of caregivers in the United States, with unique challenges due to higher mental distress and poorer physical health than noncaregivers. Latinx millennial caregivers face additional barriers, including higher rates of being uninsured and increased caregiving burdens.

**Objective:**

We used a community-informed and user-centered design approach to tailor an existing mHealth app to better meet the stress and well-being needs of Latinx millennial caregivers.

**Methods:**

We used a 2-step, multifeedback approach. In step 1, Latinx millennial caregivers participated in focus groups to evaluate wireframes for the proposed mHealth app. In step 2, participants engaged in usability testing for 1 week, concluding with short interviews for feedback. Participants were recruited through various channels, including social media and community clinics. Data were analyzed inductively using a rapid qualitative content analysis approach.

**Results:**

A total of 29 caregivers (n=20, 69% women) participated in the study. Participants had a mean age of 31 (SD 4.10) years, with most (n=28, 97%) caring for an adult and 3% (1/29) caring for children with chronic conditions. All participants completed the step 1 focus groups, with a subset of 10% (3/29) of the caregivers completing the usability testing in step 2. The most liked features included (1) the stress rating scale because it helped them understand stress and mental health; (2) the mindfulness options, which allowed for flexible timing of activities; (3) the journaling prompts for addressing daily challenges and positive experiences; and (4) the resource list for its employment and financial content. One concern was that the journaling prompts may take too much time to complete after a long and hard day. Some suggestions for improvement included a better tracking system, gamification, caregiving education, a checklist of emotions to use with the journal, tailored resources, and ways to connect with other caregivers. During step 2, participants noted that the app was user-friendly but had some glitches and unclear privacy policies. Participants liked the meditation options, resource variety, and daily stress log but wanted more journaling space, longer meditations, and additional relaxation activities.

**Conclusions:**

Future iterations should consider integrating more personalized and community-specific resources, leveraging platforms such as podcasts for broader engagement, and the use of information-based videos to support caregiver skill acquisition. Caregivers expressed needs beyond the scope of the app, such as resource access, demonstrating the need for upstream and downstream interventions. This study reinforces that user-informed design is an ongoing and iterative process that requires balancing the needs of stakeholders and the feasibility of the recommended adaptations.

## Introduction

### Background

Millennial caregivers are individuals born between 1981 and 1996 who are care providers to family members and loved ones with serious health conditions. They comprise an estimated 25% of caregivers in the United States [1,2]. The number of millennial caregivers will continue to grow as people are living longer with multiple chronic health issues [1-3]. Caring for a loved one with a chronic condition can lead to adverse physical, psychological, and social outcomes [2,4]. Millennial caregivers experience higher levels of mental distress and worsened physical health than their noncaregiver peers [3].

Millennial caregivers in the United States are a more diverse group than previous generational cohorts, with 27% identifying as Hispanic or Latinx [1]. Historically, members of the Latinx community have experienced negative determinants of health related to a lack of access to health care and health insurance [5]. These factors contribute to Latinx Americans being at higher risk of Alzheimer disease, diabetes, obesity, hypertension, and kidney disease than the general US population [4], likely increasing burdens of caregiving among the Latinx population.

Latinx millennial caregivers are at higher risk of negative health outcomes than their non-Latinx peers. The rate of uninsured Latinx citizens is more than double the rate of uninsured non-Latinx White citizens [5]. Coupled with the low rates of insurance coverage reported by millennial caregivers, Latinx millennial caregivers are at increased risk of poor health outcomes and higher rates of disease [2,5]. Latinx millennial caregivers are 34% more likely to provide a higher number of hours of caregiving than those of other races or ethnicities in the millennial caregiving population while simultaneously working more hours per week [1].

There is a dearth of research exploring interventions to enhance stress management and well-being among Latinx caregivers. The few interventions studied are highly varied and lack methods of adaptation to address the specific needs of individual caregivers [6]. Language is a significant potential barrier for this population. In the United States, 51.3% of Latinx individuals report needing translation services in health care settings, yet many trainings and public health campaigns for caregivers are only provided in English [4]. While research detailing the experiences and needs of millennial caregivers highlights the necessity for support, there is a gap regarding interventions available and tailored to this population [7,8].

Due to balancing work, family, and caregiving responsibilities, Latinx millennial caregivers may have less time to engage in coping and well-being strategies [2,7]. With the population’s needs in mind, a mobile health (mHealth) app was designed with meditation and journaling self-care exercises for the general millennial population [9,10]. The concept of an integrated mindfulness and journaling intervention delivered through an app was inspired by the dynamic fit model, which focuses on in-the-moment and long-term strategies to manage stress. Mindfulness was identified as an in-the-moment response to stress, whereas journaling would address the long-term sustained stress of caregiving [11]. A user-centered design approach modeled after the work by Still [12] was implemented to adapt the mHealth app. Feedback from the general millennial population identified the need for a resource list for caregivers and a daily stress measure in addition to mindfulness and journaling exercises. Thus, a caregiver resource list was added addressing chronic disease, work, general caregiving information, and financial information. A 1-item stress rating measure using a 10-point Likert scale was added [10]. While a resource list expanded upon the original intervention, it still remained true to the intent of supporting stress and emotional regulation. The provision of resources and information to support caregiving is a form of informational support, an important dimension of social support [13]. Social support has been found to decrease stress and burden in family caregivers [14,15].

### Objectives

The purpose of this research study was to use both community-informed and user-centered design approaches to adapt a stress and well-being app intervention for Latinx millennial caregivers during formative intervention design. The following research questions guided this study: What are Latinx millennial caregiver perspectives regarding the scope and content of the stress and well-being app intervention? What additional components or content are needed to address Latinx millennial caregiver needs? What app usability issues impact caregivers’ ability to engage with the intervention? For this adaptation, we continued to apply the approach by Still [12] coupled with a community-engaged lens [16]. Still [12] describes the importance of engaging users early and often, designing for use in context, giving users control, keeping things simple, designing for emotion, ensuring user triangulation, and discovery before designing and delivering. Wallerstein [16] describes their approach to community-based participatory research, including the contexts of research participants; partnering processes between research participants and researchers; the use of shared decision-making for research design; and outcomes that center social justice, health, and equity. This study focused on engagement rather than a full community-based participatory research approach, but we did address contexts, partnering processes, and shared decision-making throughout data collection and analysis.

## Methods

### Study Design and Participants

This was the second phase of a 3-phase multimethod study to examine the needs and experiences of Latinx millennial caregivers and adapt and refine an mHealth intervention for this population. The first phase, which focused on Latinx millennial caregivers’ needs and experiences, is reported elsewhere [7]. This second phase was focused on the adaptation and refinement of an mHealth intervention using a stepped approach. The initial qualitative descriptive analysis of focus group interviews with Latinx millennial family caregivers was followed by user testing and short interviews.

### Ethical Considerations

The focus group interviews, usability testing, and short interviews were approved by the University of Texas at Austin Institutional Review Board (STUDY00002382). Participants provided written informed consent. Confidentiality was maintained by deidentifying and storing all study data, such as notes, focus group transcripts, and data analyses, within a collaborative secure cloud storage accessible solely by research team members. Participants were provided with a US $70 gift card for taking part in the focus groups or interviews.

### Step 1

Participants were recruited using purposive sampling through multiple formats. Paid social media advertising and informal advertising within online family caregiver support group platforms was used, flyers were posted in community clinics serving low-income and uninsured individuals in Texas and Utah, and flyers were emailed to professional contacts and national caregiving groups. Finally, participants from a list from previous studies who had given permission to be recontacted were sent the study information. The eligibility survey was completed through the REDCap (Research Electronic Data Capture; Vanderbilt University) tool hosted by the University of Texas at Austin [17,18]. This survey required participants to meet the following inclusion criteria: being born between 1981 and 1996, identifying as Latinx, and providing care to a family member or friend for at least 10 hours per week. Participants were excluded if they did not have the technological capability to engage in Zoom (Zoom Video Communications) focus groups through a computer or smartphone, allowing for greater participation. According to the Pew Research Center, 93% of Latinx adults have a smartphone (similar to 91% of their White counterparts), and 75% have home broadband, with 20% of Latinx adults being dependent on a smartphone for going online. An estimated 97% of millennial adults own a smartphone, which is the age demographic for this study [19,20]. Potential participants chose the date and time of the focus group interview that would work for them, allowing for flexibility for other forms of internet access, such as through local libraries, community centers, and Wi-Fi hot spots [21].

### Step 2

A subgroup of participants was emailed about usability testing and short interviews, with an emphasis on inclusion of both Apple and Android users, gender-diverse participants, and bilingual participants to test English- and Spanish-language functionality. Initial app functionality was tested by 3 study team members before usability testing. During this portion of the study, both Latinx (n=3) and non-Latinx (n=3) millennial caregivers completed usability testing—the non-Latinx population was used as a cross-check with the Latinx population, but their data are not reported in this paper. While there is no one-size-fits-all number for usability testing, between 3 and 20 users are enough for valid results. Five to 10 users are recommended as an initial baseline for problem discovery with usability testing. The task complexity and product risk were low, so we included 3 Latinx and 3 non-Latinx users for testing [22]. For this part of the study, individuals were excluded if they did not have access to a smartphone. Participants were given instructions on how to download the app from either the Apple App Store or Google Play Store and an access code that would allow them app use for 1 week. Participants were provided with the following instructions:

- Access the phone app and use it at least 4 times per week in English and Spanish
- Access and use the meditation videos; the Stop, Take a Breath, Observe, Proceed (STOP) audio recording; and the instruction steps for loving kindness, mountain meditation, STOP, mindful breathing meditation, and journal exercises on the app at least once during the 1-week period
- Access each of the resources on the resource list on the app at least once during the 1-week period
- Complete the app reliability questionnaire at the 1-week time point
- Complete the stress measure one time during the week

It was expected that they would need to spend 1 hour over the entire week on the Uplode app to test the intervention components and app functionality.

App use data were collected on the back end to be sure that app engagement took place. Within the app, there was a link to access support from the app developer. Participants were contacted following usability testing for a short Zoom interview.

### Approach

Focusing on adaptation and refinement for the Latinx millennial caregiver population, both user-centered design and community-engaged methods were used to ensure that the functionality and content were tailored to the specific needs of this population [12,16]. Two stages of interviews with Latinx millennial caregivers were used to address stakeholder feedback, evaluate the clarity of the Spanish translation, and ensure both the usability and content tailoring of the app. In the original iteration of the mHealth intervention for millennial caregivers, cognitive interviews were used to adapt the stress and well-being mHealth intervention from a gratitude, exercise, and mindfulness intervention developed for nurses, but due to an emphasis on community engagement, we opted to use a focus group approach to support the synergy that occurs within a group dynamic [23,24].

### Data Collection

#### Step 1

Participants first indicated whether they would take part in an online focus group in the eligibility survey. Willing and eligible participants were contacted by a graduate research assistant via email, who provided participants with a link to a Zoom meeting. The principal investigator, trained in qualitative research and group facilitation, conducted 5 Zoom focus groups of 60 to 90 minutes with between 3 and 10 individuals (Textbox 1). At the end of the focus groups, participants reviewed 4 wireframes (layout of an app page that demonstrates interface components) of an mHealth app with 4 intervention components: a stress rating tool, a mindfulness activity, journaling prompts, and caregiving resources [25]. Structured questions were asked about each wireframe, and participants were encouraged to share their opinions and perspectives (Textbox 1). Before the focus groups, the principal investigator provided an introduction regarding the purpose of the focus group and ground rules for participation, including turn taking, confidentiality, and respectful communication. To avoid dominant voices within the group, individuals were cued to provide feedback for each question during the focus group interviews. Participants could build on each other’s comments and engage in group dialogue. In the case of discussion drift or a dominant voice, the principal investigator would redirect participants back to the purpose of the focus group and invite additional participation. The principal investigator used methods of deep listening, silence, probes, clarifying questions, and summarization [26]. A trained research assistant independently recorded observations in a separate document during the focus group meetings. The focus groups were recorded and transcribed verbatim. The focus groups were sequentially held until thematic saturation was achieved [27]. While the focus group interviews were offered in both English and Spanish, no participants requested Spanish interpretation during the interviews. Multiple participants identified themselves as fluent in both English and Spanish. Upon completing the focus group, participants were sent a demographic questionnaire administered through REDCap, including questions about caregiving experiences.

Textbox 1. **Interview questions.**

**Wireframe questions**

- Based on feedback from other Millennial caregivers, we have developed an app and intervention that focuses on stress, emotional regulation, and providing resources for support. We would like some feedback from each of you about what we have so far. We are sharing wireframes on the screen.
- We will start with wire frame 1, which is a stress rating scale. What do you think about this scale? Would this be helpful for you? When would it be helpful?
- We will go to wireframe 2, which is a mindfulness activity. What do you think about mindfulness? Would this be helpful for you? When would it be helpful?
- We will go to wireframe 3, which is a daily journal. It asks about what was most challenging/distressing and about what you felt most positive about for each day. What do you think about this? Would this be helpful for you? When would it be helpful?
- Now we will go to wireframe 4, which is a resource list. Looking at the headings, can you see any type of resource that would be helpful for you? Is there a type of resource that is missing?
- If you had your wish for anything to make caregiving better or easier, what would that be?
- Thank you so much for your time and participation in this study. Once again, we appreciate your contributions and the work you do as a family caregiver.

**Usability questions**

- What worked well for you with the app?
- What did not work well or was confusing?
- What did you like?
- What did you not like or what was missing?

#### Step 2

Back-end app data were collected and verified for participant use of the app. In addition, participants were asked to answer an app reliability questionnaire to identify usability issues with each app component, including mindfulness, journaling, resources, stress rating scale, and surveys. Participants engaged in a brief 15-minute Zoom interview on their experiences using the app following 1 week of use (Textbox 1). Notes were taken during these short interviews, but the interviews were not recorded or transcribed. These participants also received a gift card for their participation.

### Analysis

#### Step 1

Demographic data were analyzed for frequencies and descriptive statistics. Data from the focus group interviews were analyzed inductively using a rapid qualitative content analysis approach by the research team for coder triangulation [28,29]. An initial read-through with open coding of the interviews was conducted with 2 members of the research team to identify the main categories for matrix creation. The matrix was then tested in a coding meeting and applied to 1 focus group interview. Matrix revision was conducted, and the matrix was then applied to the rest of the focus group interviews to identify subcategories and additional main categories. Final organization of main and subcategories was conducted, including collapsing categories where appropriate. On the basis of the main and subcategories, a summary of the recommendations for changes was then reviewed with the entire team, including the app developer. A final list of revisions was created based on user and team feedback (Textbox 2). Coders and team members addressed their positionality throughout the app revision process: 2 team members identify as family caregivers, 4 identify as millennials, and 3 identify as Latinx. Discussion of identity and how this impacts data analysis and interpretation took place to ensure that team members remained close to the data and reflected what participants communicated [28]. Notes were taken during data analysis and each team meeting to serve as an audit trail [28].

Textbox 2. **App and intervention changes.**

**Changes after focus group findings**

- Spanish translation
- Caregiver support plan
- Flexibility with journaling content and amount (including emotion checklist)
- Varied mindfulness options
- Resource section: caregiving, culture, and faith; physical care tips; caregiver support groups; and state-by-state resources

**Changes after usability testing**

- App functionality issues, including Spanish-language functionality, addressed and checked by team members
- Privacy policy reviewed and addressed as a team to ensure clarity

**Future changes**

- Skill-based videos
- Podcast-like caregiver interviews
- Self-care videos
- App tailoring with activities connected to stress rating scale
- Education on cloud sharing for medication information, calendar management, and the use of a shared calendar

#### Step 2

Back-end use data were confirmed for participant use, and reliability questionnaire data were summarized and compared to those of non-Latinx millennial caregivers for similarities and differences. Open-ended response questions were analyzed along with the brief interviews using the rapid qualitative content analysis approach described for step 1. Functionality issues reported by users were relayed to the app developer.

## Results

### Overview

Results are presented sequentially by step, and participants’ quotes are shared with designation for the participant (P) and focus group (G). Changes made after each step are presented in Textbox 2. Categories and participant suggestions are outlined in Table 1.

**Table 1.** Qualitative findings.

Category and subcategory	Participant suggestions
Wireframe focus group findings	
Features that participants liked	Stress rating scale was helpful to understand mental health Multiple mindfulness options (recording and step-by-step guide) and flexibility to use them throughout the day Journaling prompts for challenges and good things may help with therapy Journal and mindfulness pages were simple and not overwhelming They liked that the integrated resources included employment-focused information
Features that participants disliked	Writing a long journal entry on difficult days or having the journal be mandatory
Recommended additions	Better tracking system over time for stress and journaling Rewards and personalized notifications within the app Tracking of stages and needs of the person they cared for, including medications and health care appointments, and having more educational information on caregiving skills Being able to select how they felt under the journaling option with a click as an alternative to writing (both positive and negative emotions) More flexibility with timing of activities Include a sleep meditation Include resources that are state specific, include more information about financial needs, and address diverse employment experiences More social options within the app to connect with other caregivers and receive answers to questions about unique needs Other stress-relieving activities such as gaming, music, in-app chatting, recipe sharing, coping with COVID-19, videos on managing stress, or other caregiver-preferred activities Connect stress rating to app activities, such as what to do with high stress levels Recommend multiple lengths of time for mindfulness (short, medium, or long)
Usability interviews	
Features that worked well	User-friendly or not overwhelming Spanish translation App was helpful when stressed
Features that did not work well or were confusing	Switching to Spanish on the app Some days, the stress log and journal did not work The meditation would stop playing when the phone went into sleep mode Unclear privacy policy Speech-to-text function did not work well
Features that participants liked	Meditation—different meditations were useful; they liked the short length Resources Daily stress log Journaling was a nice way to look back at the day
Features that participants disliked or felt were missing	Not enough space for journaling Ability to go back to the journal entries and edit them Longer meditation options More activities to help with relaxation

### Step 1

#### Overview

For step 1 of the app and intervention revision, 29 participants took part. Of these 29 participants, 20 (69%) identified as women. Participants had a mean age of 31 (SD 4.10) years, with most (n=28, 97%) caring for an adult (sibling, parent, or grandparent) and 3% (1/29) caring for children with chronic conditions.

#### Features That Participants Liked

Participants described liking the app features generally, including the stress rating scale, having multiple mindfulness options, the journaling prompts, and the resource list. Regarding the stress rating scale, they described it as being helpful in understanding stress and mental health. One participant stated that it would be “a way to keep track of what was really going on with my stress level” (P1G1). The flexibility of the mindfulness activities was noted, with P4G4 noting the importance of having options for “timing for both long and short time.” Regarding a journaling question focused on the challenges of the day and a good thing that happened that day, caregivers appreciated addressing both:

...you can kind of look back and be like okay well least this was one good thing, and this was the thing that was bad that happened, but you always have that good in the mix of it.

[P2G4]

Regarding the integrated resources, caregivers expressed interest in the employment and financial resources:

I just want to compliment you all on the financial aspect down there because you know that was something that would have been really helpful for us in the beginning.

[P1G2]

#### Features That Participants Disliked

One concern described by caregivers was having to write a long journal entry at the end of a difficult day or having the journaling questions be mandatory:

...maybe sometimes you’re too stressed and depending how my day went to be able to sit down and type a long journal may be a little bit hard for me.

[P1G1]

#### Recommended Additions

Caregivers provided multiple suggestions to improve the app both for their needs and for usability.

The need for a tracking system for stress and journaling was highlighted, including a responsive system for high stress ratings that would include app activities:

I love being able to, you know, track everything and then at the end of the month, or the end of the weekend, you can go back.

[P4G2]

Gamification, rewards, flexibility with the timing of activities, and personalized notifications were recommended to increase engagement:

...there could be a little reward encouraging to do that caregiving job.…Also if the app could have a reminder.

[P2G1]

Regarding the resource list, caregivers wanted more education about caregiving skills and space to document medication lists and health care appointments:

I think, for me, maybe like tips.…We were kind of really inexperienced with, you know, how to shower my grandfather.

[P1G2]

Caregivers suggested having a checklist of emotions rather than completing a full journal entry on the days when they were too tired or busy:

…maybe on two days I can just maybe click on how I’m feeling.

[P1G1]

Other stress-relieving activities were recommended, such as gaming, music, recipe sharing, videos for managing stress, and information on coping with COVID-19:

Space to add some music...I do love classical music.

[P2G1]

They also described the need for multiple lengths of time for mindfulness (“timing for both long and short” [P4G4]) and a sleep meditation (“like a night meditation” [P1G4]).

Regarding the resource list, caregivers described wanting tailored resources specific to their state, work situation, or caregiving needs:

...having it more where you can actually look for your particular state, because each state kind of offers different benefits.

[P2G4]

Caregivers wanted more connection with other caregivers for “a community to share problems” (P2G2) and to receive advice:

...it would be nice, knowing that there are other people like me, too, and we could share useful info and advice.

[P3G5]

### Step 2

#### Overview

After app revisions were made (outlined in Table 1), a subsample (3/29, 10%) completed usability testing and included 67% (2/3) women and 33% (1/3) men. General millennial caregivers (n=3) who were fluent in English and Spanish completed usability testing concurrently, although only findings from Latinx caregivers are reported in this paper. App use, reliability questionnaire, and qualitative findings are outlined for each step in the following sections and in Table 1.

#### App Use and Reliability

All 3 participants completed the requested activities in English, and 67% (2/3) of the participants completed them in Spanish unless there were reported functionality issues associated with an activity reported through the app reliability questionnaire. One participant could not complete the activities in Spanish, although during the interview, this issue was clarified, with the participant not understanding how to switch from English to Spanish. The main functionality issues reported through the questionnaire were with loading the mindfulness and journaling activities. Similar issues arose for both Latinx and non-Latinx millennial caregivers. One user reported timing out of the mindfulness recordings and videos and one activity not loading at all (3 events). One participant noted the stress rating scale not recording on one day and the journaling activity not loading one day. No other functionality issues were reported.

#### Features That Worked Well

One participant described the app as user-friendly, “easy to implement,” and not overwhelming. In total, 67% (2/3) of the participants felt that the Spanish translation worked well. One participant described the app as being helpful when they were stressed.

#### Features That Did Not Work Well or Were Confusing

For one participant, switching to Spanish did not work. During the interview, the issue was clarified, and the participant was able to then switch to Spanish. Participants noted a few glitches, such as the stress log or journal not working on some days, the meditation not working while in sleep mode, and the speech-to-text function for journaling not working well within the app. One participant described the privacy policy for the app as unclear.

#### Features That Participants Liked

Participants again described liking most dimensions of the app, including the meditation length and variety, the resource variety, the daily stress log, and the use of the journal for reflection on the day.

#### Features That Participants Disliked or Felt Were Missing

Participants recommended having more space for journaling, the ability to edit journal entries, longer meditation options, and more activities to help with relaxation.

## Discussion

### Principal Findings

In this study, we sought to integrate community- and user-informed design into our adaptation of an mHealth stress and well-being intervention for Latinx millennial family caregivers. The original intervention was developed for the general millennial caregiver population. Significant differences we noted in the needs and preferences between groups (general millennial caregiver population and the Latinx millennial family caregiver population) were the inclusion of other stress-relieving activities beyond mindfulness and journaling, having an option not to journal and just indicate their emotions, and having tailored employment and financial resources for their specific situations and locations that extended the resources already embedded in the app [7,8,10]. Similar to general millennial users, the Latinx users identified the need for social engagement and community [7,10].

This 2-step process of adapting and refining an mHealth app–delivered intervention for Latinx millennial family caregivers revealed the need to expand resources, such as state- and community-focused resources that address financial and employment needs. Religious and cultural information, state resources, and support groups were added to the resource list to address the need for more resources and social connection. A caregiver support plan informed by safety plans for individuals at risk of suicide [30] was created. The mHealth app and intervention were also translated into Spanish to address language access [31]. This expansion of social support resources remains aligned with the original intent of the intervention to support stress and well-being [13-15]. The addition of the emotion checklist provides more of the in-the-moment response to stress that is found with mindfulness, emphasizing awareness of the present moment and acknowledgment of one’s emotions [32]. There may be a limitation for some users who only use in-the-moment responses with mindfulness and an emotion checklist versus those who have a sustained response to stress through journaling [11]. For Latinx millennial caregivers, there is an important balance in adapting interventions to align with their busy schedules while also providing a therapeutic and research- and theory-informed approach to intervention delivery [1,8,33]. With future research, we can assess whether differences in use among the emotion checklist, journaling, or both impact caregivers’ stress and well-being outcomes.

We recognize that gaps remain regarding the desire for community and having resources that are tailored to the individual. This might call for a layered intervention approach that leverages mHealth and individual or group-based caregiver coaching (either in-person or remote coaching) to address these additional needs [34]. This might also require artificial intelligence solutions to pull in comprehensive, tailored, and location-based resources, such as the PaidLeave.AI platform developed by Moms First [35]. Indeed, most mHealth apps and interventions focused on family caregivers have limited functionality; thus, there is a need for multifunctional and adaptable mHealth interventions for this population [36].

While mHealth interventions have the benefit of being accessible and time-limited [37], which works well for the millennial caregiver population [38], they often do not solve larger system issues such as resource access, social isolation, navigation, and advocacy that can help this group of Latinx caregivers who are already navigating system-level barriers and power structures [39,40]. This calls for upstream interventions, which were largely outside the scope of this study but could be considered for future intervention iterations as findings and resources expand. This tightrope of addressing user feedback while not overstretching resources is a well-acknowledged challenge in product development and technology-based interventions [41]. A strength of this specific study was ensuring that stakeholder needs were addressed and shared decision-making took place [16].

Considerations for changes in the future include leveraging podcast delivery methods to share caregiver stories and skill-based and self-care content. The podcast platform is widely used by millennials and has been found to support health outcomes such as behavior change and social interaction [42,43]. For Latinx individuals specifically, the podcast platform is a growing medium [44]. Caregivers have anecdotally and in formal reports expressed the desire for a one-stop shop platform that addresses financial or employment, educational, navigation, shared care, and self-care needs [45]. Further iterations could explore the feasibility of these options [36]. One danger is the platform becoming unwieldy, difficult to use, and less reflective of stakeholder-reported needs, thereby violating the principles of simplicity and shared decision-making. Therefore, continued stakeholder feedback will be required [12,16].

Despite noted gaps in our app-based intervention currently, we believe that there are important future opportunities to address Latinx millennial family caregiver well-being. The population interviewed in this study was in early and middle adulthood, a critical life phase for adult development; they experience health disparities related to system-level issues regarding insurance, health care access, and economic opportunities, and they are navigating caregiving while also balancing work, family, and other social responsibilities [1,2,4,5]. Thus, their risks of long-term health consequences related to family caregiving are high, and there continues to be a gap in interventions tailored to this specific group [6]. It is important to intervene early with this population to address these health consequences that may influence their life trajectories. With our app-based intervention, we hope to meet caregivers where they are in an accessible format to support their well-being. Future research will not only continue to address cultural, contextual, and usability issues regarding this mHealth intervention but also address its feasibility, acceptability, and efficacy.

### Limitations

Our study had important limitations that should be considered when interpreting our findings. First, we had an overrepresentation of female Latinx millennial family caregivers. In the Latinx millennial population, the gender split is almost even [1,2]. Due to our emphasis on a user-informed process, this overrepresentation of women could have affected our findings and subsequent intervention and app adaptations. Interestingly, this gender split is reflective of a broader trend in the literature, with women engaging more with mHealth interventions than men [46]. Future research steps should implement recruitment measures that address these gender inequalities in mHealth research. In addition, our usability testing sample of Latinx millennial caregivers was small but within the recommended parameters for usability testing. This size is adequate for problem testing but may not have been adequate for additional usability issues involving a broader and more diverse sample. We also used a disease-agnostic approach, which means that our intervention and app were not tailored to the specific disease process that these caregivers were supporting. Further iterations could address more tailored applications for disease-related issues. Finally, our sample only included participants with access to a computer or smartphone for focus group participation and a smartphone for usability testing. While the percentage of Latinx individuals in the United States with a smartphone is high (93%), there remains a proportion of the population that would have been excluded from the study [20]. This population would be more likely to be of a low income. This affects the generalizability of our findings. In future work, provision of smartphones with data plans would overcome this gap.

### Conclusions

With our user-informed process, we adapted a stress and well-being intervention for Latinx millennial family caregivers. During this process, we noted the difficulty of aligning the study purposes and resources with stakeholder feedback. We acknowledge that user-informed design is an ongoing and iterative process that requires balancing the needs of stakeholders and the feasibility of the recommended adaptations. We anticipate ongoing intervention and mHealth app adaptations that address more stakeholder-identified needs, more thoroughly address the need for social connection, and are individually tailored.

## Abbreviations

mHealth: mobile health
REDCap: Research Electronic Data Capture
STOP: Stop, Take a Breath, Observe, Proceed

## References

[1] Flinn B (2018). Millennials: the emerging generation of family caregivers. Innov Aging.

[2] (2020). Caregiving in the US 2020. https://www.aarp.org/content/dam/aarp/ppi/2020/05/full-report-caregiving-in-the-united-states.doi.10.26419-2Fppi.00103.001.pdf.

[3] Reed N, Bouldin E, Taylor C, McGuire L (2020). Millennials as caregivers: results from the BRFSS, 44 states, DC, and Puerto Rico, 2015-2018. Innovation in Aging.

[4] Sehar U, Rawat P, Choudhury M (2023). Comprehensive understanding of Hispanic caregivers: focus on innovative methods and validations. J Alzheimers Dis Rep.

[5] (2021). Health insurance coverage and access to care among Latinos: recent trends and key challenges. https://aspe.hhs.gov/reports/health-insurance-coverage-access-care-latinos.

[6] McCarthy MJ, Sanchez A, Garcia YE, Bakas T (2021). A systematic review of psychosocial interventions for Latinx and American Indian patient-family caregiver dyads coping with chronic health conditions. Transl Behav Med.

[7] Cleary C, Dupree G, Welling A (2024). Experiences and supportive care needs of Latinx millennial caregivers. J Transcult Nurs.

[8] Thomas Hebdon MC, Jones M, Neller S (2022). Stress and supportive care needs of millennial caregivers: a qualitative analysis. West J Nurs Res.

[9] Gallagher VT, Reilly SE, Martin D, Manning C, Shaffer KM (2024). Examining differences in health-related technology use between millennial and older generations of caregivers. Nurs Rep.

[10] Hebdon M, Thomas M, Peterson NE (2023). User-centered adaptation of an mhealth intervention for stress and emotional regulation in millennial caregivers. J Inform Nurs.

[11] Harkness K, Hayden EP (2018). The Oxford Handbook of Stress and Mental Health.

[12] Still B (2017). Fundamentals of User-Centered Design: A Practical Approach.

[13] Health behavior and health education | Part three, chapter nine: measuring constructs social support. University of Pennsylvania.

[14] Ou PR, Wu MH, Tsai ST, Ma YC (2024). The relationship between social support and stress in family caregivers of stroke patients. J Neurosci Nurs.

[15] Bongelli R, Busilacchi G, Pacifico A (2024). Caregiving burden, social support, and psychological well-being among family caregivers of older Italians: a cross-sectional study. Front Public Health.

[16] Wallerstein N (2021). Engage for equity: advancing the fields of community-based participatory research and community-engaged research in community psychology and the social sciences. Am J Community Psychol.

[17] Harris PA, Taylor R, Thielke R, Payne J, Gonzalez N, Conde JG (2009). Research electronic data capture (REDCap) - a metadata-driven methodology and workflow process for providing translational research informatics support. J Biomed Inform.

[18] Harris PA, Taylor R, Minor BL (2019). The REDCap consortium: building an international community of software platform partners. J Biomed Inform.

[19] Gelles-Watnick R (2024). Americans’ use of mobile technology and home broadband. Pew Research Center.

[20] (2024). Demographics of mobile device ownership and adoption in the United States. Pew Research Center.

[21] (2024). Five resources to help newcomers access affordable internet. Switchboard.

[22] Six JM, Macefield R (2016). How to determine the right number of participants for usability studies. UXmatters.

[23] Focus groups. Community Commons.

[24] Or CK, Holden RJ, Valdez RS, Duffy VG, Ziefle M, Rau PL, Tseng MM (2023). Human-Automation Interaction: Mobile Computing.

[25] What is wireframing?. Experience UX.

[26] University of Kansas Toolkit for conducting focus groups. Community Tool Box.

[27] Saunders B, Sim J, Kingstone T (2018). Saturation in qualitative research: exploring its conceptualization and operationalization. Qual Quant.

[28] Lincoln YS, Guba EG (1985). Naturalistic Inquiry.

[29] Nevedal AL, Reardon CM, Opra Widerquist MA (2021). Rapid versus traditional qualitative analysis using the Consolidated Framework for Implementation Research (CFIR). Implement Sci.

[30] Stanley B, Brown GK (2012). Safety planning intervention: a brief intervention to mitigate suicide risk. Cogn Behav Pract.

[31] Escobedo LE, Cervantes L, Havranek E (2023). Barriers in healthcare for Latinx patients with limited English proficiency - a narrative review. J Gen Intern Med.

[32] Kabat-Zinn J (2005). Full Catastrophe Living: Using the Wisdom of Your Body and Mind to Face Stress, Pain, and Illness.

[33] Lachman ME (2004). Development in midlife. Annu Rev Psychol.

[34] Santarossa S, Kane D, Senn CY, Woodruff SJ (2018). Exploring the role of in-person components for online health behavior change interventions: can a digital person-to-person component suffice?. J Med Internet Res.

[35] Paid Leave.

[36] Park JYE, Tracy CS, Gray CS (2022). Mobile phone apps for family caregivers: a scoping review and qualitative content analysis. Digit Health.

[37] Dugas M, Gao GG, Agarwal R (2020). Unpacking mHealth interventions: a systematic review of behavior change techniques used in randomized controlled trials assessing mHealth effectiveness. Digit Health.

[38] Vogels EA (2019). Millennials stand out for their technology use, but older generations also embrace digital life. Pew Research Center.

[39] Velasco-Mondragon E, Jimenez A, Palladino-Davis AG, Davis D, Escamilla-Cejudo JA (2016). Hispanic health in the USA: a scoping review of the literature. Public Health Rev.

[40] Veinot TC, Ancker JS, Cole-Lewis H (2019). Leveling up: on the potential of upstream health informatics interventions to enhance health equity. Med Care.

[41] Integrating user feedback without overextending resources. FasterCapital.

[42] Amador FLD, Alves GCG, Santos VRD, Moreira RSL (2024). Use of podcasts for health education: a scoping review. Rev Bras Enferm.

[43] Miller J (2016). Who listens to podcasts–and why they matter to marketers. LinkedIn.

[44] Gonzalez DR (2023). Spanish language podcasts: a gateway for Latino Audiences in the U.S. LinkedIn.

[45] In their own words: family caregivers from across the country share their priorities and recommendations. National Academy for State Health Policy.

[46] Szinay D, Forbes CC, Busse H, DeSmet A, Smit ES, König LM (2023). Is the uptake, engagement, and effectiveness of exclusively mobile interventions for the promotion of weight-related behaviors equal for all? A systematic review. Obes Rev.

